# Association Study Identifying a New Susceptibility Gene (*AUTS2*) for Schizophrenia

**DOI:** 10.3390/ijms151119406

**Published:** 2014-10-24

**Authors:** Bao Zhang, Yue-Hong Xu, Shu-Guang Wei, Hong-Bo Zhang, Dong-Ke Fu, Zu-Fei Feng, Fang-Lin Guan, Yong-Sheng Zhu, Sheng-Bin Li

**Affiliations:** College of Forensic Science, Health Science Center, Xi’an Jiaotong University, Xi’an 710061, China; E-Mails: zhangbao_814@mail.xjtu.edu.cn (B.Z.); yueyuehong921@stu.xjtu.edu.cn (Y.-H.X.); weisg@mail.xjtu.edu.cn (S.-G.W.); zhanghb@mail.xjtu.edu.cn (H.-B.Z.); fdk.1991.02.07@stu.xjtu.edu.cn (D.-K.F.); zufeifeng@mail.xjtu.edu.cn (Z.-F.F.); fanglingguan@mail.xjtu.edu.cn (F.-L.G.); zys3000@mail.xjtu.edu.cn (Y.-S.Z.)

**Keywords:** *AUTS2* gene, SNP, schizophrenia, association study

## Abstract

Schizophrenia (SCZ) is a severe and debilitating mental disorder, and the specific genetic factors that underlie the risk for SCZ remain elusive. The autism susceptibility candidate 2 (*AUTS2*) gene has been reported to be associated with autism, suicide, alcohol consumption, and heroin dependence. We hypothesized that *AUTS2* might be associated with SCZ. In the present study, three polymorphisms (rs6943555, rs7459368, and rs9886351) in the *AUTS2* gene were genotyped in 410 patients with SCZ and 435 controls using polymerase chain reaction-restriction fragment length polymorphism (PCR-RFLP) and forced PCR-RFLP methods. We detected an association between SCZ and the rs6943555 genotype distribution (odds ratio (OR) = 1.363, 95% confidence interval (CI): 0.848–2.191, *p* = 0.001). The association remained significant after adjusting for gender, and a significant effect (*p* = 0.001) was observed among the females. In the present study, rs6943555 was determined to be associated with female SCZ. Our results confirm previous reports which have suggested that rs6943555 might elucidate the pathogenesis of schizophrenia and play an important role in its etiology.

## 1. Introduction

Schizophrenia (SCZ) is a severe and debilitating mental disorder with a prevalence of approximately 1% in the general worldwide population [1]. SCZ is globally considered to be the most serious form of mental illness. Not only does SCZ incur psychological and physiological suffering, but it also adversely affects public security. Despite the continuing efforts to identify the etiologies of SCZ, the definite causes of this disorder remain unclear [2,3]; However, epidemiological studies have demonstrated that SCZ is a familiar disorder with a complex mode of inheritance and a heritability that is as high as 80% [4].

Recently, many genome-wide association studies (GWAS) have detected new evidence related to the genetics of SCZ by focusing on single-nucleotide polymorphisms (SNPs) and copy number variation (CNV) [5,6,7,8], and the results have been consistently replicated in association studies [9,10,11,12]. Although GWA studies have provided a promising approach for the study of the genetics of complex diseases, they have not explained the majority of the underlying genetic risks for SCZ. Thus, the identification of individual candidate genes with small effects on disease risks remains important [13].

The autism susceptibility candidate 2 (*AUTS2*) gene maps to 7q11, spans 1.2 Mb, includes 19 exons, and encodes a nuclear protein that is expressed primarily in the developing brain [14]. Although the function of this protein remains unclear, many studies had revealed that *AUTS2* is highly expressed in the neural tube and the embryonic, fetal and adult brain; these findings indicate that *AUTS2* might play important roles during neuronal development, and variations of this gene might affect transcriptional regulation and be related to neurodevelopmental diseases [14,15,16].

Many studies have shown that *AUTS2* is associated with autism [17,18,19], alcohol consumption [20], heroin dependence [21] and suicide [22]. The following findings are particularly noteworthy: (1) the same chromosomal rearrangements and several single genes, such as the contact in associated protein-like 2 (*CNTNAP2*) gene, have emerged as genetic risks for autism and SCZ [23]; (2) one study found that SCZ in males and females is associated with 10- and 20-fold increases, respectively, in the risk of suicide and that these risks increase to 30- and 140-fold among men and women, respectively, under the age of 35 years [24]; and (3) one study reviewed the evidence that developmental neuropathologies in hippocampal and prefrontal cortical pathways contribute both to the symptoms of SCZ and to the vulnerability to addictive behavior via dysfunctional interactions with the nucleus accumbens. Thus, drug addiction and SCZ might have overlapping neurobiological substrates within the hippocampus and subcortical structures [25,26,27]. Taken together, these findings indicate that it is vital to determine whether *AUTS2* is also associated with SCZ. To test this hypothesis, we conducted a genetic association study in a sample from the Han population.

## 2. Results

The three genetic variations of the *AUTS2* gene were successfully identified in 410 SCZ cases and 435 controls using polymerase chain reaction-restriction fragment length polymorphism (PCR-RFLP) or forced PCR-RFLP in the present study. No significant deviations from Hardy–Weinberg equilibrium (HWE) were found in the cases or controls. The allelic and genotypic frequencies of the three SNPs were shown in Table 1. The estimated risks associated with these polymorphisms in the SCZ patients and the healthy controls were tested according to three models (*i.e.*, dominant, recessive and addictive models) that were also illustrated in Table 1. We performed association analyses for every identified SNP. When all of the samples were considered, the genotype association analysis of SNP1 (rs6943555) under the dominant model revealed a significant result (odds ratio (OR) = 1.363, 95% confidence interval (CI): 0.848–2.191, *p* = 0.001).

**Figure 1:**
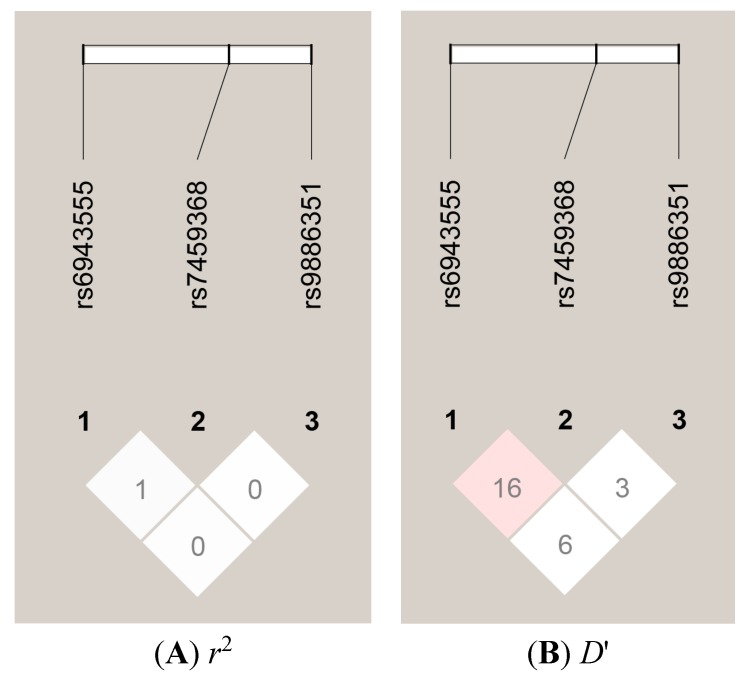
Linkage disequilibrium (LD) plots of the three single-nucleotide polymorphisms (SNPs) of the autism susceptibility candidate 2 (*AUTS2*) gene. The values in the squares are the pair-wise calculations of *r*^2^ (**A**) or *D*' (**B**). The white squares with the “0” indicate *r*^2^ = 0 (*i.e.*, No LD between a pair of SNPs). The white squares with the “16” indicate *D*' = 0.16 (*i.e.*, very low LD between a pair of SNPs).

Linkage disequilibrium (LD) estimations for the three SNPs of the *AUTS2* gene were performed. We observed that three SNPs exhibited very low *D*' and *r*^2^ values, which suggested that calculations of haplotype frequencies were not required (Figure 1). To examine whether gender influenced this association, we analyzed our data separately for the males and females based of the above results. After dividing the sample according to sex, we found that, among the females, rs6943555 AA homozygotes were over-represented among the SCZ subjects compared to the control subjects (*p* = 0.001) in females (Table 2).

**Table 1:** Allelic and genotypic frequencies of the single-nucleotide polymorphism (SNP) association analyze.

Makers		Allele Frequency (%)		*p*-Value ^a^	OR ^b^ (95% CI)	Genotype Frequency (%)			*p*-Value ^a^	HWE *p*-Value	Models	OR ^b^ (95% CI)	*p*-Value ^a^
SNPs	ID												
SNP1	rs6943555	A	T	0.097		AA	AT	TT	0.001	0.496			
SCZ		33.0	77.0		1.191	7.8	50.5	41.7			Dominant	1.363 (0.848–2.191)	**0.001**
CTR		29.3	70.7		(0.969–1.463)	10.4	37.9	51.7			Recessive	0.734 (0.456–1.180)	0.201
											Addictive	1.197 (0.971–1.475)	0.093
SNP2	rs7459368	G	A	0.720		GG	AG	AA	0.809	0.954			
SCZ		17.4	82.6		1.047	2.7	29.5	67.8			Dominant	1.117 (0.495–2.523)	0.809
CTR		16.8	83.2		(0.813–1.349)	3.0	27.6	69.4			Recessive	0.895 (0.396–2.021)	0.789
											Addictive	1.048 (0.813–1.351)	0.719
SNP3	rs9886351	G	A	0.681		GG	AG	AA	0.803	0.114			
SCZ		27.4	72.6		0.956	8.3	38.3	53.4			Dominant	1.028 (0.632–1.672)	0.803
CTR		26.6	73.4		(0.771–1.185)	8.5	36.1	55.4			Recessive	1.067 (0.662–1.719)	0.789
											Addictive	1.030 (0.825–1.286)	0.794

SCZ: schizophrenia; CTR: control; OR: odds ratio; CI: confidence interval; SNP1: rs6943555; SNP2: rS7459368; SNP3: rS9886351; HWE: Hardy–Weinberg equilibrium. Significant *p*-values and HWE *p*-values were shown in bold font. a: *p*-values were based on normal chi-square statistics; b: OR refers to the risk allele odds ratio for both the cases and controls.

**Table 2:** Sex-specific allele and genotype association analyze.

Marks and Sex		Allele Frequency (%)		*p*-Value ^a^	OR ^b^ (95% CI)	Genotype Frequency (%)			*p*-Value ^a^
SNP1		A	T			AA	AT	TT	
F	SCZ	35.0	65.0	0.284	1.251 (0.881–1.538)	8.3	53.4	38.3	**0.001**
	CTR	30.0	70.0			12.3	35.4	52.3	
M	SCZ	31.1	68.9	0.399	1.140 (0.84–1.547)	7.8	41.1	51.0	0.223
	CTR	28.4	71.6			7.4	47.5	45.1	
SNP2		G	A			GG	AG	AA	
F	SCZ	17.7	82.3	0.453	0.874 (0.615–1.242)	3.4	28.6	68.0	0.641
	CTR	15.8	84.2			2.1	27.6	70.4	
M	SCZ	17.2	82.8	0.764	1.058 (0.733–1.525)	2.0	30.4	67.6	0.395
	CTR	18	82.0			4.2	27.6	68.2	
SNP3		G	A			GG	AG	AA	
F	SCZ	30.1	69.9	0.119	0.791 (0.589–1.062)	9.2	40.8	50.0	0.283
	CTR	25.1	74.9			7.5	35.3	57.2	
M	SCZ	25.2	74.8	0.318	1.174 (0.857–1.608)	7.3	35.8	56.9	0.596
	CTR	28.4	71.6			9.9	37.0	53.1	

SCZ: schizophrenia; CTR: control; SNP1: rs6943555; SNP2: rS7459368; SNP3: rS9886351; F: female; M: male. Significant *p*-values are shown in bold font. a: *p*-values were based the normal chi-square statistics. b: OR refers to the risk allele odds ratio for both the cases and controls.

## 3. Discussion

The purpose of this study was to investigate the association between the rs6943555, rs7459368, and rs9886351 polymorphisms of the *AUTS2* gene and the presence of SCZ. Three SNPs were genotyped by PCR-RFLP or forced PCR-RFLP methods, the latter of which is a very useful, accurate and easy-to-handle strategy that overcomes the weaknesses of complicated technical demands, slow speeds and unstable reproducibilities [28].

We found that rs6943555 of the *AUTS2* gene was strongly associated with SCZ and that the rs6943555 AA genotype was associated with a slight increase in the risk of SCZ. Furthermore, when the data were divided according to sex, a significant association was observed between rs6943555 and SCZ in the female population. To the best of our knowledge, this is the first report of a significant association between an *AUTS2* gene polymorphism and SCZ. The following lines of evidence suggested that this observed association was unlikely to be an artifact. First, both the findings regarding rs6943555 in the entire samples and the sex-specific association analyses support the association. Second, our subjects were from the same geographical region and this geographic matching served as a good proxy for genetic matching.

The *AUTS2* gene is a novel gene that is initially linked to neurodevelopmental disorders in a pair of autistic monozygotic twin sisters [29]. Following the report of this finding, many studies have found structural variants that disrupt the *AUTS2* region and are related to intellectual disabilities and developmental delays [30,31]. Additional structural variants of *AUTS2*, some of which are intronic, have also been shown to be associated with attention deficit hyperactivity disorder (ADHD) [32] epilepsy [33,34], dyslexia [30], language delay, visual impairment, microcephaly and other conditions. Interesting, one recent study identified the intronic SNP rs6943555 in the fourth intron of the *AUTS2* to be the SNP that is most strongly associated with alcohol consumption [20]. Furthermore, another study verified that rs6943555 is significantly associated with heroin dependence [21]. Previous studies have revealed that drug addiction and SCZ might have overlapping neurobiological substrates within the hippocampus and subcortical structures, which suggests that SCZ patients are at an increased risk of developing substance use disorders [26,27]. Conversely, individuals at elevated risks for substance use disorders might be particularly vulnerable to SCZ [35]. The direction of the observed association between rs6943555 and SCZ observed in this study is consistent with associations with heroin dependence [21] and suicide committed under influence of ethanol [22] that have been reported previously. The OR for the rs6943555 A allele in relation to SCZ was 1.191 in our data, and other studies have reported ORs of 1.24 for heroin dependence [21] and 1.17 for suicide under the influence of ethanol [22]. Furthermore, we found that the AA homozygote of rs6943555 was significantly over-represented in the SCZ subjects (*p* = 0.001), which is similar to the finding that has been reported for heroin-dependent subjects (*p* = 0.017).

Interestingly, we compared the allelic frequencies of rs6943555 with those from HapMap [36] and found that the frequencies in the control subjects were lower than those in the HapMap HCB (HapMap Han Chinese in Beijing) population (0.29 in the current sample and 0.37 in the HapMap HCB population). This difference might be due to the difference in the sample size of the HapMap database (*n* = 78) and our sample size (*n* = 435). Although there might not be a simple interpretation of these differences, more reliable data regarding the SNPs in the Han Chinese population will be obtained in future studies.

There are several limitations to this study. Although rs6943555 was associated with SCZ in the present study, it remains unclear whether this is a primary association or one of the effect markers of the *AUTS2* gene. It is also possible that multiple variants in *AUTS2* had independent effects on SCZ. Because we tested only three small LD markers, we were unable to answer this question. The cross-sectional nature of this study precluded the establishment of causality. Future research is needed to replicate our findings with longitudinal analyses. Moreover, the small SCZ sample size might have limited the detection of weaker relationships in volume, but we found clear between-group differences with respect to shape abnormalities. Thus, future research is needed to examine whether additional SNPs of the *AUTS2* gene are associated with SCZ.

## 4. Materials and Methods

In the present study, the sample included 410 patients with SCZ (204 males and 206 females, mean age: 35.2 ± 11.4 years) and 435 healthy controls (192 males and 243 females, mean age: 37.6 ± 10.8 years) who enrolled from the Mental Health Center and Medical Examination Center of First Affiliated Hospital, Xi’an Jiaotong University. Experienced psychiatrists assessed each subject using the Structured Clinical Interview for DSM-IV Axis I Disorders (SCID). Detailed information, including the presence of mental retardation, personality disorders, and a brief description of the subject’s psychosocial and occupational functioning during the course of the illness, were recorded for SCZ diagnoses. Patients with SCZ were interviewed independently by two experienced psychiatrists to ensure that they met the Diagnostic and Statistical Manual of Mental Disorders, 4th revision (DSM-IV) criteria for SCZ. These were interview assessments of personal histories, hospital records, and family-history reports. Research subjects with diagnoses of substance-induced psychotic disorders, learning disabilities, head injuries, epilepsy, mood disorders, mental retardation or other symptomatic psychoses were excluded from this study. The controls were confirmed to lack any mental illness and were matched with patients in terms of origin, sex, age, and education level.

All participants were volunteers and longstanding residents of Shanxi province and provided written informed consent prior to inclusion. The study protocol was approved by the institutional review board of the Xi’an Jiaotong University College of medicine, with project identification code (2011-054).

### 4.1. Single-Nucleotide Polymorphism (SNP) Selection

The rs6943555 SNPs of the *AUTS2* gene have been reported to be associated with alcohol consumption at a genome-wide level of significance (*p* = 4 × 10^−8^ to *p* = 4 × 10^−9^) [20], heroin dependence (*p* = 0.017) [21], and suicide (*p* = 0.018) [22]. In the current study, rs6943555 was selected as the candidate SNP. Furthermore, we conducted preliminary analysis using the HapMap data to select rs6943555-adjacent tagSNPs. The selected adjacent tagSNPs met the following criteria [37]. First, we examined tagSNPs in Haploview (v4.2) using the CHB population and a minor allele frequency cut-off (MAF) > 5%. Second, a MAF > 20% with pair-wise tagging and *r*^2^ > 0.8 [35] were used as cut-off for the selection of tagSNPs. Thus, rs7459368 and rs9886351 were selected as the tagSNPs in blocks that were adjacent to rs6943555. In total, three SNPs, *i.e.*, rs6943555, rs7459368 and rs9886351, were genotyped in this study.

### 4.2. Genotyping

PCR primers were designed according to the NCBI database (NC_000007.14). Three polymorphisms in the *AUTS2* gene were genotyped with PCR-RFLP or forced PCR-RFLP methods, and one of the primers contained one- or two-nucleotide mismatches to enable us to use restriction enzymes to discriminate sequence variations [28]. The primers, selected restriction enzymes (Fermentas, Thermo Scientific, Beijing, China), and fragment sizes are given in Table 3.

**Table 3:** Primers used to identify genetic variants of the *AUTS2* gene.

SNPs	Primers (5'–3')	Sizes (bp)	T (°C)	Enzymes/Regions	T (°C)
SNP1	F: 5'-TGGGTGTTGGAAGAGTTTTGA-3'; R: 5'-ATACAGTATACATAAACATTGGAAAAGAGG***G***AA-3'	196	60	Hinf1, G▼ANTC	37
SNP2	F: 5'-AAAGTTCTGGACAGTGGTGCTC-3'; R: 5'-TTCTGACAGTGCGTAAAGGTTG-3'	257	65	Msp1, C▼CGG	37
SNP3	F: 5'-GGTGGAAAATAAGCCAGTATGC-3'; R: 5'-TAGGAAAATGGATTAAACGTAGG***A***G-3'	221	65	Hinf1, G▼ANTC	37

SNP: single nucleotide polymorphism; SNP1: rs6943555; SNP2: rs7459368; SNP3: rs9886351; SNP1: ***G*** is the mismatched base; SNP3: ***A*** is the mismatched base.

The 15-μL PCR reaction volumes contained the following: 50 ng genomic DNA, 10 pmol of each primer, 7.5 μL SuperMixTaq (RUNDE, Shaanxi, China), ddH_2_O (produced by our lab) to reach a volume of 15 μL. The PCR protocol was 95 °C for 5 min followed by 35 cycles of 95 °C for 30 s, annealing of each primer pair at 60 °C/65 °C/65 °C for 30 s, 72 °C for 30 s and a final extension at 72 °C for 10 min. After the PCR reaction, aliquots of 10 μL PCR product from each pair of primers were digested with 10 U Hinf1 (SNP1)/Msp1 (SNP2)/Hinf1 (SNP3) for 8 h at the same temperature of 37 °C (restriction enzymes including Hinf1 and Map1 were from Fermentas, Thermo Scientific, Beijing, China). The digested products were detected by electrophoresis and were stained with ethidium bromide after approximately 1–2 h in 3% agarose gel (GENE TECH, Shanghai, China).

### 4.3. Statistical Analyses

The statistical power of our sample size was calculated with the G*Power program (Franz Faul, University Kiel, Kiel, Germany) according to Cohen’s method [38]. Our sample size exhibited >80% power for the detection of significant (*p* < 0.05) associations among the genotypes, alleles and haplotypes at an effect size index of 0.1 (corresponding to a “weak” gene effect).

In the present study, the Hardy–Weinberg equilibrium (HWE) of the genotype counts was tested with SHEsis (Yong YONG, Shanghai Jiao Tong University, Shanghai, China) [39].The differences in the frequencies of the alleles, genotypes and haplotypes between the cases and controls were evaluated with chi-square analyses. The genotypes, haplotypes and SCZ associations were estimated by computing the odds ratios (ORs) and their 95% confidence intervals (CIs) using logistic regression. Pair-wise linkage disequilibrium (LD) statistics (*D*' and *r*^2^) and haplotype frequencies were computed with Haploview 4.2 (Harvard University, Boston, MA, USA) [40] to construct haplotype blocks and to evaluate deviations from HWE. The haplotype blocks were defined according to the criteria of Gabriel [41]. Furthermore, stratified analyses were conducted to detect whether differences in genes and gender influenced any such associations. All statistical analyses were performed using SPSS 18.0 software (SPSS Inc., Chicago, IL, USA).

## 5. Conclusions

Our findings suggested that rs6943555 of the *AUTS2* gene is associated with SCZ in Han Chinese patients and indicate that the *AUTS2* gene might play an important role in the etiology of SCZ. Given the complex patterns offindings from association studies that have focused on SCZ and its underlying genetic heterogeneity, further studies and replications with larger samples, particularly samples of different ethnic groups, are required.
